# Sterility and structural variation in an arabidopsis pedigree carrying a ring minichromosome

**DOI:** 10.1007/s10577-025-09776-0

**Published:** 2025-08-01

**Authors:** Benny Ordoñez, Weier Guo, Witsarut Chueakhunthod, Isabelle M. Henry, Luca Comai

**Affiliations:** 1 Plant Biology and Genome Center, University of California, 1 Shields Avenue, Davis, CA 95616 USA; 2 Present address: San Joaquin Valley Agricultural Center, United States Dept of Agriculture, Parlier, CA 93648 USA; 3 Horticulture and Agronomy Graduate Group, Department of Plant Sciences, University of California, 1 Shields Avenue, Davis, CA 95616 USA; 4 Department of Horticulture, Faculty of Agriculture, Kasetsart University, 50 Ngam Wong Wan Rd., Lat Yao, Chatuchak, 10900 Bangkok Thailand

**Keywords:** Genome instability, Centromere, Circular DNA, Deletion, Fitness, Supernumerary chromosome, DNA junction mapping, Mitotic stability, Meiotic transmission

## Abstract

Circular minichromosomes could be useful tools for plant biotechnology, yet their long-term structural stability, heritability, and effects on phenotype remain poorly understood. In this study, we report a multi-generational analysis of the Arabidopsis *mini1a* ring minichromosome, which originated from the chromosome 1 centromere in a haploid induction cross. Is *mini1a* unstable, as suggested by classical studies on other ring chromosomes? Using whole-genome sequencing of individuals carrying *mini1a* representing multiple successive generations, we uncovered a major catastrophe driven by DNA breaks and novel junction formation, resulting in a new version of *mini1a,* that carries a 1.3 Mb deletion in the centromeric region (*mini1a*Δ). We identified 20 new breakpoints, of which 7 disrupted gene bodies—a frequency unlikely to occur by chance. Interestingly, both *mini1a* and *mini1a*Δ could exist in one or two copies and could co-exist in a single plant. Although they were inherited efficiently, their presence was sometimes associated with plant sectors with 100% sterility. These findings highlight the structural plasticity of *mini1a.* At the same time, they raise questions regarding the mechanisms underlying the observed reduced plant fertility. In summary, circular minichromosomes can be deleterious and biotechnology applications based on the manipulation of minichromosomes will require careful planning and testing.

**Supplementary Information:**

The online version contains supplementary material available at 10.1007/s10577-025-09776-0.

## Introduction

Minichromosomes or ‘minis’, are small chromosomes that can emerge spontaneously from instability in larger, normal chromosomes. These elements have been observed in multiple plant species, including Arabidopsis, maize, tobacco, barley, and potato (Murata et al. 2006; Murata 2014; Yan et al. 2017; Yin et al. 2021).

Notwithstanding multiple studies, formation and stability of these small chromosomes over successive generations remains unclear. Specifically, understanding their long-term stability, structural dynamics, and potential influence on phenotype and karyotype evolution is crucial, as minichromosomes could serve as platforms for genetic engineering and crop improvement, for example by providing stacks of genes affecting important traits to multiple well-adapted varieties without the need for recombination (Birchler 2015; Gambogi et al. 2024). In addition, minichromosomes could fuel karyotypic variation through the formation of novel chromosomes and thus participate in genome evolution.

Minis can emerge through various processes, including X-ray mutagenesis, biolistic methods, recombination systems like Cre/LoxP, Agrobacterium-mediated transformation, wide hybridization, and genome elimination (Kapusi et al. 2012; Birchler and Han 2018; Liu et al. 2022). Their formation likely involves multiple processes, including non-homologous end-joining (NEHJ), homology-directed repair (HDR), chromosome missegregation, breakage-fusion-bridge (BFB) cycles, and chromoanagenesis (McClintock 1931; Birchler and Han 2013; Comai and Tan 2019; Guo et al. 2023).

In Arabidopsis, minichromosomes have been reported in the output of genome elimination crosses involving centromere-specific histone 3 (CENH3)-based haploid induction (Ravi and Chan 2010). For instance, (Seymour et al. 2012) observed three double haploid lines carrying minichromosomes in the diploidized progeny of such crosses. Formation of minis in haploid progeny was reported by (Kuppu et al. 2015), confirming their formation early during haploid induction. A potential mechanism was provided by Tan et al. (2015), who demonstrated extensive chromoanagenesis, i.e. chromosomal restructuring, including shattered chromosomes derived from the haploid inducer (HI) genome among the aneuploid offspring. A fragment spanning the centromere could circularize and form small rearranged chromosomes (Guo et al. 2023). Tan et al. (2023) further demonstrated that positive selection using a transgenic marker linked to the centromere could be used to identify haploids that carried a mini chromosome. Taken together, these findings indicate that approximately 1% of phenotypically normal *Arabidopsis* haploids carry rearranged small chromosomes originating from centromeric and adjacent pericentromeric regions.

Tan et al. (2023) analyzed the cytological and genetic features of a 7 Mb minichromosome named *mini1a* formed from CEN1 of Arabidopsis Col-0. They observed that, at prophase and metaphase of meiosis I, *mini1a* does not pair with the homologous full-size chromosome, but displays a circular structure instead, with a distinct heterochromatic region. *Mini1a* was present in one or two copies. In many crosses, its transmission rate resembled that of a trisomic chromosome, but it varied considerably across generations and in some cases failed to transmit entirely. The cause of this behavior remained unknown. In summary, the use of haploid induction crosses followed by molecular or genetic screens can systematically generate minichromosomes in a controlled manner. It is likely that similar minis may be isolated in crop species where haploid induction through genome elimination is possible. Because many of these minis include few genes, minichromosomes are expected to have a minimal impact on phenotype and should aid experiments aimed at plant genome engineering.

Instability of minis could result from their structure. Circular chromosomes, for example, can be destabilized by crossover between sister chromatids (McClintock 1932). Recombination forms dicentric or concatenated structures that break upon anaphase leading to subsequent break-fusion-bridge (BFB) cycles. In this study, we evaluate the fate of *mini1a* across generations in Arabidopsis. We focus on four key aspects: (1) the copy number stability of *mini1a*, (2) the timing and nature of structural rearrangements, (3) the identification and timing of novel DNA junctions, and 4) the effect of the presence of *mini1a* on phenotype. In short, we show that *mini1a* can exist in one or two copies, that each copy number state can be associated with either the original form or with an alternative rearranged version, that both forms of *mini1a* can be transmitted efficiently, and likely, that they can undergo further, relatively frequent somatic changes of an unknown nature that result in normal growth, but lead to complete sterility. Our results enhance our understanding of minichromosome fate and constitute important information for their application in plant breeding and genetic engineering.

## Material and methods

### Plant material and growth conditions

The Arabidopsis line *mini1a* was derived from a haploid induction cross between the SQ-8 and NFA-8 ecotypes, using the GFP-tailswap haploid inducer in the Columbia (Col-0) background. One of the resulting plants carried a truncated chromosome 1 (Tan et al. 2023), derived from the haploid inducer, a strain engineered in the Col-0 background. Tan et al.(2023) also performed cytological and genomic characterization of the *mini1a* line, providing insights into its circular structure and inheritance. The mini was formed in the background of an SQ-8/NFA-8 F2 hybrid. Subsequent selfing advanced homozygosity. The resulting lines, therefore, carry the mini derived from Col-0 chr.1 in an SQ-8/NFA-8 recombinant inbred background.

In this study, we used plants from generations 5 to 10 (S5 to S10) of the *mini1a* line. We surface-sterilized the *mini1a* line seeds with 20% (v/v) bleach and vernalized them for 3 days at 4 °C. Then, we either planted the seeds in mixed soil or germinated them on Murashige and Skoog medium supplemented with 1% (w/v) agar, adjusted to pH 5.6. Afterward, we transferred them to a growth chamber at 22 °C for 5–6 weeks, under a 16-h light/8-h dark cycle.

### Mini1a structure variation across generations assessment

In a previous study (Tan et al. 2023), the presence of *mini1a* was primarily detected through PCR of the junction site. While this approach effectively identified the presence of *mini1a*, it may have overlooked structural variation within the mini. To address this limitation, we conducted a comprehensive analysis of structural variation in *mini1a* across generations S5 to S10, utilizing short-read whole-genome sequencing. The seed-mother plant of each generation was no longer available to us (Fig. S1). Accordingly, we sowed and grew seedlings that were siblings of the seed-mother plant. Individuals were randomly selected for sequencing in each generation, and PCR was performed to detect the *mini1a* junction. The final cohort of 46 lines included both *mini1a*-carrying and junction-negative individuals: S5 (n = 3), S6 (n = 8), S7 (n = 11), S8 (n = 11), S9 (n = 10), and S10 (n = 3) (Table 1).

**Table 1:** Mini1a Types and Sequenced Lines Across Generations (S5-S10)

Generation	Sequenced lines	*Mini1a* types
S5	1S5N, 2S5Y*, 3S5Y	1 × full *mini1a*
S6	5S6N, 7S6N, 9S6Y*, 10S6Y, 11S6Y*, 12S6Y*, 13S6Y*, 17S6Y	2 × deleted *mini1a*, 2 × deleted *mini1a*, 1 × deleted *mini1a*, no *mini1a*
S7	18S7Y, 19S7N*, 20S7N*, 21S7N, 22S7N, 23S7N, 24S7Y, 25S7N, 26S7N,27S7Y, 28S7Y	no *mini1a*
S8	29S8Y*, 30S8N, 31S8Y, 32S8Y, 35S8N, 36S8N, 37S8N, 40S8N, 42S8Y*, 44S8Y, 45S8Y	1 × full *mini1a*, 2 × full *mini1a*
S9	50S9N, 51S9Y, 52S9Y*, 53S9Y, 54S9N, 55S9Y*, 57S9Y, 58S9Y, 59S9Y*	2 × deleted *mini1a*, 2 × deleted *mini1a*, 2 × deleted *mini1a*
S10	61S9Y, 63S9N, 64S9N	no *mini1a*

Codes represent the individual number, generation, and PCR status for *mini1a* presence (e.g., 3S5Y indicates individual 3 from generation S5, PCR-positive for *mini1a* presence). Resequenced lines are marked with an asterisk

We included individuals lacking a detectable *mini1a* junction in our sequencing dataset because we hypothesized that a subset of PCR-negative samples could represent false negatives. Such cases may arise from structural rearrangements of the mini or loss of the junction site, both of which could prevent PCR detection despite the continued presence of *mini1a*.

For each selected individual, we extracted genomic DNA (gDNA) as previously described (Ghislain et al. 2004), and prepared libraries using 400 bp sheared input DNA with KAPA Hyper Prep kit (cat. No KK8504). Sequencing was performed by Novogene (Novogene Inc) to obtain 150 PE reads. Raw reads were trimmed using Trim_galore (version 0.6.7, https://github.com/FelixKrueger/TrimGalore) with Cutadapt (version 3.4). We aligned the short reads to the Col-CEN reference genome (Naish et al. 2021) using the Burrows-Wheeler Aligner (BWA) (version 0.7.17) with default parameters (Li 2013).

We conducted dosage variation analysis along non-overlapping consecutive bins spanning the entire genome, following the method outlined in (Henry et al. 2015). Briefly, we normalized bin coverage using a customized script (https://github.com/Comai-Lab/bin-by-sam) to one of the individuals from the same pedigree that did not carry *mini1a*. We set the expected relative read coverage for a diploid individual to 2, while values of 1 or 3 indicate deletion or duplication, respectively.

### Mini1a rearrangement analysis

To understand the structural dynamics of *mini1a* across generations, we analyzed novel DNA junctions in selected *Arabidopsis* lines. We resequenced 12 lines from the S5-S10 generations, including 2S5Y, 9S6Y, 11S6Y, 12S6Y, 13S6Y, 19S7N, 20S7N, 29S8Y, 42S8Y, 52S9Y, 55S9Y, and 59S9Y, using Illumina paired-end reads at a depth of 26x. We then merged the newly generated reads with previously sequenced data. To detect novel DNA junctions formed by reorganized genomic fragments, we followed the methods of Guo et al. (2021). Briefly, we searched for sequencing reads that aligned to two genomic regions at least 2000 bp apart on the reference genome (Col-CEN). We used a custom Python script (https://github.com/guoweier/Poplar_Chromoanagenesis) to locate genomic regions containing the specified reads.

We established two thresholds to minimize false positives. The first threshold stipulates that novel junctions must only exist in samples carrying *mini1a*, and be completely absent in the controls. The second threshold stipulates a minimum coverage for the cross-junction reads. Minimum coverage was calculated as follows: we divided the genome into non-overlapping, consecutive 5 kb bins, treating the boundaries between consecutive bins as pseudo-junctions. For each pseudo-junction, we calculated the average read coverage and divided it by 2, as the Arabidopsis genome is diploid and expected to contain two copies of these pseudo-junctions while only one copy is anticipated to be affected by the novel junctions.

### Validation of novel potential DNA junctions

We employed a custom Python script (https://github.com/guoweier/Poplar_Chromoanagenesis) to extract the cross-junction reads identified at the selected bins from each line's alignment file (SAM file) to construct putative novel junctions. We then constructed contigs spanning these novel junctions using PRICE assembly (Ruby et al. 2013). To confirm the sequence of these novel junctions, we compared the output contigs with the Col-CEN reference genome's sequence (Naish et al. 2021) using a custom bash script (https://github.com/guoweier/Poplar_Chromoanagenesis). A new junction was considered validated in silico when both ends of the contig aligned with the expected regions.

We further validated the underlying new junctions through PCR and subsequent Sanger sequencing. Primers were designed using Primer3 (Li and Durbin 2009) and are detailed in File S1. For each potential junction, we designed six primer pairs: two pairs located on the chimeric contig, two pairs in the surrounding regions, and two positive control pairs. PCR was performed in a 10 μL reaction volume, which included 2X GoTaq Green Master Mix (Promega Corporation, Madison, WI), 1 ng sample gDNA, and 0.5 μM of each forward and reverse primer. Reactions were held at 95 °C for 2 min, followed by 35 cycles of denaturation at 95 °C for 30 s, annealing at 58 °C for 30 s, and extension at 72 °C for 1 min and 20 s, concluding with a final extension at 72 °C for 5 min. We separated the PCR products on a 1% agarose gel and purified them using the QIAquick Gel Extraction Kit (Qiagen) for Sanger sequencing, which was performed by Azenta Life Sciences.

### Enrichment ratio analysis

The Arabidopsis genome annotation file, including the genomic locations of genes and repeat-masked data (Col-CEN_v1.2), was obtained from https://github.com/schatzlab/Col-CEN. A custom Python script was used to compute the density of genomic features around breakpoints (https://github.com/guoweier/Poplar_Chromoanagenesis). To determine whether novel breakpoints displayed significantly different enrichment ratios from random genomic positions, we performed a one-sample t-test. For the samples, a window with customized size was centered on each breakpoint, and the nucleotides associated with targeted features were counted. For constructing the control population, we randomly selected 10,000 pseudo-breakpoints located at Chr01 13 Mb-20 Mb regions, and calculated the mean value of their feature density. This analysis was performed 1,000 times, and finally generated a distribution of feature density. The enrichment ratio was calculated as:

$$Enrichment \;Ratio=\frac{Mean \;density \;of \;real \;breakpoints}{Mean \;density \;of\; pseudo-breakpoints}$$

With sample set and population set, the one-sample t-test was performed and the significance was determined (p-values < 0.05).

### Pollen viability

For the pollen viability assays, we selected five anthers from individuals carrying the *mini1a* minichromosome across generations S5 to S8 as follows: S5 (n = 4), S6 (n = 1), S7 (n = 3), and S8 (n = 5). From each individual, we sampled three flower buds from two branches. As a control, we also collected flower buds from individuals without *mini1a* in S5 (n = 1), S6 (n = 4), S7 (n = 1), and S8 (n = 2). We stained the anthers with Alexander dye and examined them under light microscopy, following the protocol described by (Alexander 1969). This method differentiates viable pollen grains, which retain a red cytoplasm, from non-viable grains, which appear green due to cytoplasmic degradation, providing a clear assessment of pollen viability.

## Results

### Mini1a disrupts male fertility

To assess the phenotypic consequences of *mini1a*, we analyzed male fertility in plants across four generations (S5–S8), comparing *mini1a*-carrying individuals with non-*mini1a* control siblings from the same generations. The only consistent phenotypic difference was reduced fertility, evidenced by the small, seed-depleted siliques in some branches.

We examined ten anthers per individual, selecting five anthers from flower buds on each of two different branches, and assessed pollen viability using Alexander staining. In all non-*mini1a* carrier plants (n = 8), viability was uniformly high, with an average of 100% viable pollen per anther across all generations. In contrast, *mini1a*-carrying individuals (n = 13) exhibited a wide range of pollen viability, with per-plant averages ranging from 10 to 70%. For example, in generation S5, *mini1a* carriers showed viability between 30 and 68%; in generation S7, between 0 and 52%; and in generation S8, between 10 and 70%. The only *mini1a*-carrying individual from generation S6 displayed a moderate average viability of 60%, while its non-carrier siblings were all fully fertile.

**Fig. 1:**
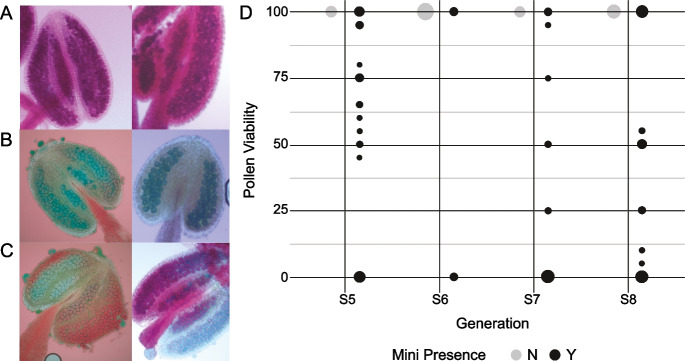
**Pollen viability of *mini1a* lines**. **a-c**) Alexander's staining of anthers from plants carrying *mini1a* from generation S7. Wild-type pollen stains red while the aberrant pollen grains stain green. a) Anthers displaying fully viable pollen, **b**) Anthers devoid of viable pollen, **c**) Anthers showing variability in pollen viability depending on sectors and **d**) Pollen viability percentage distribution in individuals that inherited *mini1a* and individuals that did not. Dot size represents the number of individuals characterized in each category. For each generation, grey dots correspond to control individuals that do not carry *mini1a* while black dots correspond to individuals that carry at least one copy of *mini1a*

Among the anthers examined, we observed an unusual phenotype. Typically, anthers display uniformly red pollen grains (indicating viability) or, occasionally, green pollen grains (indicating non-viability), as seen in both fertile and sterile individuals (Fig. 1A, B). However, some anthers in *mini1a* carriers exhibited sharply defined viable and non-viable sectors, a striking phenotype absent in non-*mini1a* individuals (Fig. 1C). This sectoring pattern was observed in two individuals from generation S5 and one from generation S7. These findings are summarized in Fig. 1D, which presents the distribution of pollen viability across all analyzed plants.

These results demonstrate that while *mini1a* can be transmitted without disrupting fertility, it is frequently associated with partial or complete male sterility. This dichotomy demonstrates the potential for *mini1a* to undergo catastrophic events affecting reproductive success.

### Structural stability and copy number variation of mini1a are variable across generations

**Fig. 2:**
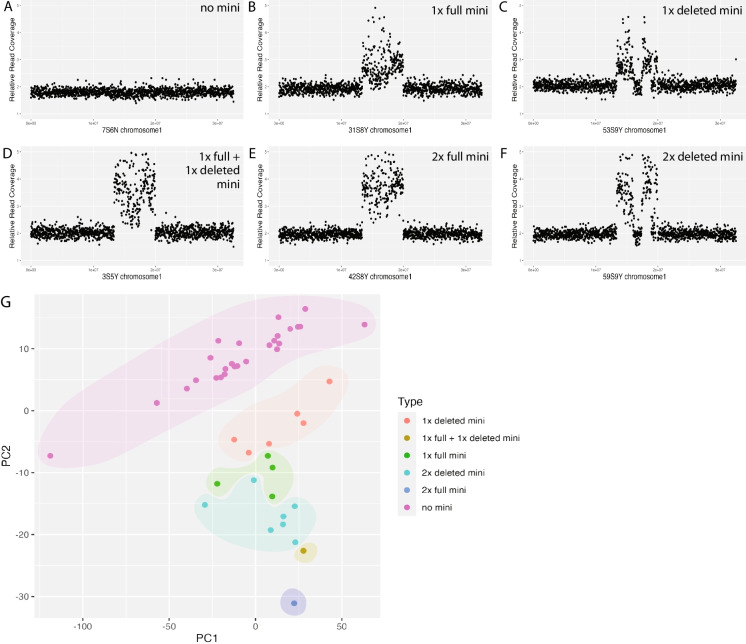
Minichromosomes types. (**A**) Principal Component Analysis (PCA) of Arabidopsis lines with and without mini1a, based on copy number variation across the mini1a region. Individuals fall into 6 categories: 1 × deleted mini (orange), 1 × full + 1 × deleted mini (brown), 1 × full mini (green), 2 × deleted mini (cyan), 2 × full mini (blue), no mini (magenta). (**B-G**) Dosage plot of chromosome 1 for the six mini1a types, generated using Illumina short-read sequencing data. Reads were pooled into non-overlapping consecutive 25 kb bins and normalized to a control individual that does not carry mini1a to derive relative read coverage

To assess the structural stability and copy number of *mini1a* across generations, we performed Illumina sequencing at approximately 10X coverage on 46 individuals from generations S5 to S10. Reads were mapped to the Col-CEN reference genome (Naish et al. 2021), which provides high continuity assembly of centromeric arrays. The coverage was calculated for non-overlapping 25,000 bp bins (Fig. 2A-F). Because this analysis was performed several years after the original pedigree was built, we were unable to sequence the seed-mother of each generation. Instead, we grew and sampled siblings of each seed-mother plant (Fig. S1).

The *mini1a* minichromosome spans a region from 13 to 19 Mb and was previously shown to be circular by junction sequencing and cytology (Tan et al. 2023). This includes the active, CENH3-defined centromere (15 to 17 Mb) (Naish et al. 2021). We used PCR amplification of the junction region to detect the presence of the mini. We conducted a closer examination of the sequence read coverage in the affected segment and in the surrounding areas. Using the coverage data binned on 100 kb intervals, we performed a pattern-based cluster PCA of the *mini1a* lines, which revealed six distinct structural patterns: (1) one full copy of *mini1a*, (2) two full copies of *mini1a*, (3) a deleted version of *mini1a*, (4) two copies of the same deleted version of *mini1a,* (5) the presence of one full copies and one deleted version of *mini1a*, and (6) complete loss of *mini1a* (Fig. 2G). These patterns provided a detailed illustration of the overall structural variation present in *mini1a* across generations.

**Fig. 3:**
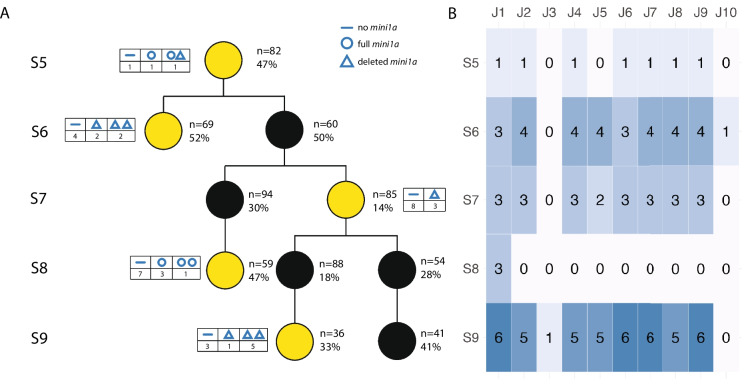
**Pedigree and inheritance of the minichromosome**. (**A**) Pedigree of Arabidopsis mini1a lines from generations S5 to S9. Yellow circles represent groups for which at least one individual was sequenced while, black circles represent groups that were not sequenced. In all cases, sequencing started after the pedigree reached the S9 and the true parents of each generation were no longer available. For this reason, siblings of each parental plant were sequenced (Fig. S1). The numbers and percentage next to each circle indicate the number of individuals in the group and the frequency of mini1a inheritance. The tables next to yellow circles indicate the mini1a types found in each group (see Fig. 2 for details): a line represents absence of mini1a; a blue circle represents a full size mini1a; a blue triangle represents mini1aΔ. Lines with 2 minichromosomes are shown with 2 corresponding symbols. (**B**) Heatmap of the junctions detected in each generation. The column headings represent the 10 validated junctions, the y-axis indicates S5-S9 generations, as depicted in (**A**). The numbers in the table cells represent the number of individuals in the sequencing group with the corresponding junctions

In Generation S5, among the three individuals assessed, one carried one full copy of *mini1a*, one harbored one full copy as well as a single deleted version of *mini1a* (referred to as *mini1a*Δ hereafter), and one did not carry any *mini1a* (Fig. 3). The single deletion pattern observed in the S5 remained largely consistent in subsequent generations, indicating stable inheritance of this structural variant. Both *mini1a* and *mini1a*Δ could result in sterility (compare Fig. 1 with Fig. 3). In each generation, we identified individuals harboring zero, one or two copies of *mini1a*. *Mini1a* dosage was distinctly single or double and never intermediate, which suggested that the two-copy state was mitotically stable. Importantly, individuals that tested negative for *mini1a* by PCR never showed evidence of *mini1a* sequences in the sequencing data, confirming the absence of *mini1a* in these lines. Among the individuals positive for *mini1a* or *mini1a*Δ, at least four lines exhibited additional smaller rearrangements, which could be further classified by analyzing breakpoint regions (see below). By Generation S7, the pedigree clearly separated lines carrying *mini1a* from those carrying *mini1a*Δ.

Taken together, these findings indicate that a major deletion (~ 1.3 Mb) affecting *mini1a* likely occurred in the early generations. Both forms of the mini can coexist in a plant because the dosage pattern of one individual in S5 indicated the presence of both *mini1a* and *mini1a*Δ. Further, both the S6 and S7 progenitor plants (which were not sequenced directly) produced progeny with one or the other form and, therefore, carried both forms. We conclude that both types could be inherited through selfing. Previous cytological studies (Tan et al. 2023) provided no evidence of pairing, an infrequent property of small chromosomes (Han et al. 2007). We assume, therefore, that two minis partition randomly to meiotic products.

### Novel DNA junctions reveal structural evolution of mini1a

To further investigate the structural dynamics of *mini1a* across generations, we identified novel DNA junctions associated with *mini1a*. We resequenced 12 of the previously sequenced lines from different generations (S5 to S9) and acquired Illumina paired-end reads with a depth of 26 × after merging old and new sequenced reads (Table 2). The merged reads from all samples, except 13S6Y, displayed *mini1a* patterns similar to those observed in the old reads alone. We then performed junction detection as described in the Methods.

**Table 2:** List of the 12 lines that were resequenced and their *mini1a* types

Sample ID	*mini1a* Type
2S5Y	1 × full *mini1a*
9S6Y	2 × deleted *mini1a*
11S6Y	2 × deleted *mini1a*
12S6Y	1 × deleted *mini1a*
13S6Y	no *mini1a*
19S7N	no *mini1a*
20S7N	no *mini1a*
29S8Y	1 × full *mini1a*
42S8Y	2 × full *mini1a*
52S9Y	2 × deleted *mini1a*
55S9Y	2 × deleted *mini1a*
59S9Y	2 × deleted *mini1a*

**Fig. 4:**
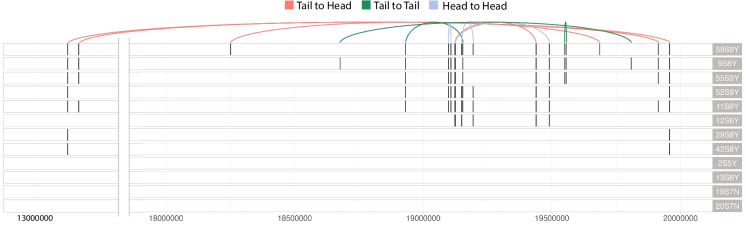
Distribution of validated DNA junctions along Chromosome 1. The X-axis represents 13 Mb—20 Mb on Chromosome 1. Each track represents one Arabidopsis line. The black vertical lines represent validated breakpoints. The curves on top connected 2 breakpoints that were joined together in restructured samples. Red lines represent breakpoints joined head-to-tail, green lines represent breakpoints joined tail-to-tail, and blue lines represent breakpoints joined head-to-head

We identified 25 potential novel DNA junctions in silico, and validated 10 of them through Sanger sequencing of PCR amplified junctions (Fig. 3B and File S2). Of these 10 junctions, 8 appeared in multiple *mini1a* Arabidopsis lines, while the remaining 2 were unique to a single sample (File S2). One junction corresponded to the circularization site (Tan et al. 2023). These junctions displayed different fragment-joining orientations with no preferential arrangement (p-value = 0.9, Chi-square test). In all 10 cases, both breakpoints were located within the *mini1a* region (Chr01:13-19 Mb) (Fig. 4). We did not identify the junction corresponding to the large deletion in the centromeric array. This is probably due to the difficulty in mapping short reads to the array. We used SNPs between the parents and the *mini1a* sequence to identify the haplotype carrying these novel junctions. For 11 of the breakpoints, we confirmed that the surrounding sequence was of Col-0 origin, indicating they were part of the *mini1a* haplotype. The remaining 9 breakpoints lacked SNPs required to distinguish between the parental and *mini1a* genomes (File S3). Taken together, only 2 junctions could be between *mini1a* and a normal chr.1, but all other data confirm that these novel junctions appeared on the Col-0 *mini1a* haplotype.

Of these 10 junctions, 8 were consistently associated with the *mini1a*Δ variant (deleted mini chromosome). For example, in S5, there is one sample carrying one copy of full mini (*mini1a)* and one copy of deleted mini (*mini1a*Δ)*,* and all the novel junctions were observed on this sample (Fig. 3 and File S2). Samples in S6, S7 and S9 only carried deleted mini (*mini1a*Δ) showed more junction presence, while samples in S8 only carried full mini (*mini1a*) carried only junction 1, which resulted from the initial circularization event (Fig. 3 and File S2).

To investigate whether these novel breakpoints occurred preferentially in gene or non-genic space, we calculated the enrichment ratio of gene density and repeat density around those breakpoints. Since breakpoints are all located in the centromeric region, we only used the centromeric region as the control. Detection of breaks in repeated regions is challenging and our discovery was likely biased. Statistical analysis revealed genes might be enriched around validated breakpoints, but only the analysis involving the 10 kb window displayed significance (p-value = 0.01) (Table 3). Repeats were depleted around validated breakpoints, but neither 1 kb nor 10 kb window analysis was statistically significant (Table 3). Among the 20 breakpoints, 7 directly disrupted gene bodies—a frequency unlikely to occur by chance (p of random frequency = 0.0124). The 7 genes that were directly affected were shown to be involved in a range of biological functions such as photosynthesis, root nutrient absorption and reproductive development (File S4).

**Table 3:** Enrichment ratios of junction breakpoints detected in *mini1a* compared to typical centromeric regions

Genomic features	# of junction	1 kb window		10 kb window	
		**Enrichment ratio**	**P-value**	**Enrichment ratio**	**P-value**
Gene	10	1.28	0.42	1.65	0.01*
Repeats	10	0.63	0.06	0.77	0.08

Enrichment ratio calculation was described in Material and Methods. The value > 1 represents higher than control; the value < 1 represents lower than control. *: P-value < 0.05

## Discussion

We examined the structural dynamics and phenotypic impact of *mini1a* across multiple generations in Arabidopsis, elucidating their stability and heritability. The *mini1a* chromosome originated in a haploid induction cross aimed at elimination of the Col-0 chromosomes. Following chromoanagenesis, a centromere-containing fragment of chr.1 circularized (Tan et al. 2023). Although our analysis could not investigate the actual seed-mother plants of each generation (Fig. S1, See Methods), analysis of their siblings provided considerable insights. At generation S5, when our sampling started, we found two forms of the mini-chromosome: the original and one containing a large deletion called *mini1a*Δ. The 10 novel junctions identified in *mini1a*Δ in the S5, persisted through S9. Therefore, the structural rearrangements occurred at or before the S5. The rearrangements include a ~ 1.3 Mb deletion spanning from 16 to 17.3 Mb of chr 1. The active CEN1 defined by CENH3 ChIP spans from 15 to 17 Mb (Wlodzimierz et al. 2023). Therefore, loss of half of CENH3 chromatin did not impair the transmission of *mini1a*Δ (Fig. 3) suggesting that half of the centromeric arrays are sufficient for segregation or that the active centromere of *mini1a*Δ spread to the neighboring repeats and expanded to a size comparable to that of the wild-type CEN1.

**Fig. 5:**
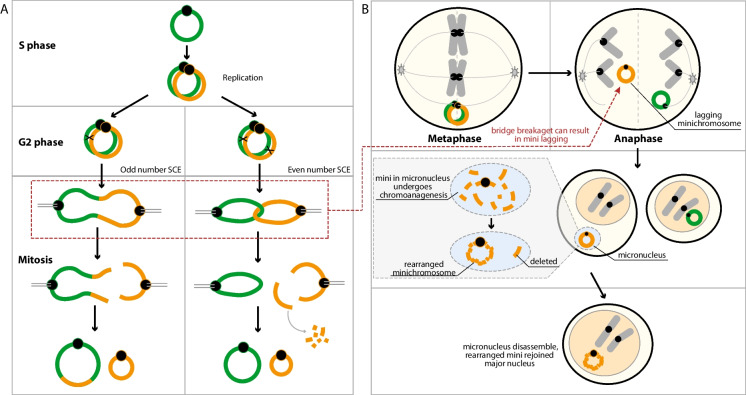
**Two potential mechanisms of minichromosome instability** (**A**) The fate of a ring mini through the cell cycle depends on sister chromatid exchange (SCE) during the S and G2 phases. Ring minis remain intact in the absence of SCE. When there is an odd number of SCEs, ring chromosomes double in size and form dicentric chromosomes. In the case of an even number of SCEs, chromosomes either separate normally or form interlocked rings, which break during the metaphase-anaphase transition, producing rings or fragments of different sizes. This illustration is based on (Pristyazhnyuk and Menzorov 2018) (2018). (**B**) The ring minis can also lag during cell division, and become trapped into a micronucleus. Later, during the next cell cycle, the mini inside micronucleus can undergo chromoanagenesis, a catastrophic pulverization and reorganization event, forming a complex rearranged mini with some pieces deleted. When the micronucleus disassembles, the rearranged mini rejoins the major nucleus, and it can stably be transmitted into the next generation

When did *mini1a*Δ originate? A dosage plot of DNA isolated in the S2 generation does not display the deletion (Tan et al. 2023). Therefore, we infer that *mini1a*Δ arose from genome instability between the S2 and S5 generation. The different changes could have occurred episodically. However, the lack of substantial new changes in the S5-S9 generations is inconsistent with chronic instability and suggests that a single catastrophic event resulted in multiple changes. Circular chromosomes are prone to breakage-fusion-bridge (BFB) instability resulting from crossover between chromatids and leading to concatenation or to fusion and dicentric formation (Fig. 5A) (McClintock 1932). Interestingly, we did not observe further significant changes in generations S5 to S10. This does not mean that either type of minichromosome has achieved stability. The other possible mechanism is chromoanagenesis (Fig. 5B). According to this hypothesis, a mis-segregated *mini1a* formed a micronucleus at telophase, underwent catastrophic fragmentation and reorganized as *mini1a*Δ, Subsequently, *mini1a*Δ was restituted into the major nucleus. The relative rarity of missegregation and chromoanagenesis could explain why *mini1a*Δ did not show significant structural changes in S5-S10.

An unexpected finding with either *mini1a* forms, was frequent male sterility, visible as sectors of anthers or affecting entire secondary shoots (Fig. 1). These sporadic but recurring events suggest that a mitotically heritable change associated with the minichromosome engenders male sterility. What could be the nature of this change? A disrupting allelic dosage linked to the presence of the mini-chromosome is possible, as some genes are present in three copies, but it is not clear how this could result in the observed sectors. On the other hand, sectoring could result from additional imbalance caused by duplication of genes within *mini1a*. Another candidate genetic change is chromosomal translocation which, when heterozygous, is known to induce sterility in mammals and plants (Lyon and Meredith 1966; Sakamoto et al. 2017). A dsDNA break in *mini1a* could lead to recombination with chr.1 or with other chromosomes in a manner similar to that of McClintock’s factor X, a minichromosome based on chr.9 in maize (Birchler and Han 2018). Another possible change could be epigenetic. Genes on the mini may become silenced (Hung and Slotkin 2021) and repress essential meiotic or gametophytic genes. These mechanisms, however, are speculative and require further investigation.

In conclusion, the characterization of *mini1a* inheritance has led to interesting findings. First, we are unaware of any previous reports connecting sterility to a circular chromosome. This property, while potentially idiosyncratic to *mini1a*, deserves further characterization. If controlled and limited to anthers, it may facilitate crop hybridization. While incompatible with seed-based field productivity, it does not result in an obvious vegetative phenotype suggesting meiotic or postmeiotic action. Accordingly, use in crop species of an engineered minichromosome with similar properties might be compatible with biomass, leaf, or tuber production. Extended clonal stability, which could not be explored in Arabidopsis due to its short life, should be examined in a clonal plant. Second, the observed centromeric remodeling in *mini1a*Δ indicates dsDNA breaks occurred in the centromeric repeat arrays. It demonstrates both fragility and plasticity of the mini centromere and, because *mini1a* is dispensable, it could be used to analyze the effect of deletions on centromere function and transmission (Kaszás and Birchler 1998). In contrast to the behavior of this ring chromosome, linear minichromosomes are mitotically very stable (Birchler 2015). Third, the minichromosome was inherited by a substantial fraction of the progeny and could exist in one or two copies, which could be readily differentiated by sequencing read dosage analysis (Fig. 2A-F). In conclusion, our analysis indicates that circular minichromosomes can be deleterious and reduce fitness. At the same time, they are sufficiently stable to be employed in basic studies and, after careful characterization, may still be useful biotechnological tools.

## Supplementary Information

Below is the link to the electronic supplementary material.

Supplementary file1 (DOCX 363 KB)

## Data Availability

The sequence reads used in this analysis are available at https://www.ncbi.nlm.nih.gov/bioproject under BioProject PRJNA1296752.
