# Supplementary material for: Sterility and structural variation in an arabidopsis pedigree carrying a ring minichromosome
Source: Chromosome Res. 2025 Aug 1;33(1):16. doi: 10.1007/s10577-025-09776-0 (PMC12316722; doi:10.1007/s10577-025-09776-0)
Supplement: Supplementary file 1 — Supplementary file1 (DOCX 363 KB) [file 10577_2025_9776_MOESM1_ESM.docx]

## Supplementary Figures and Files


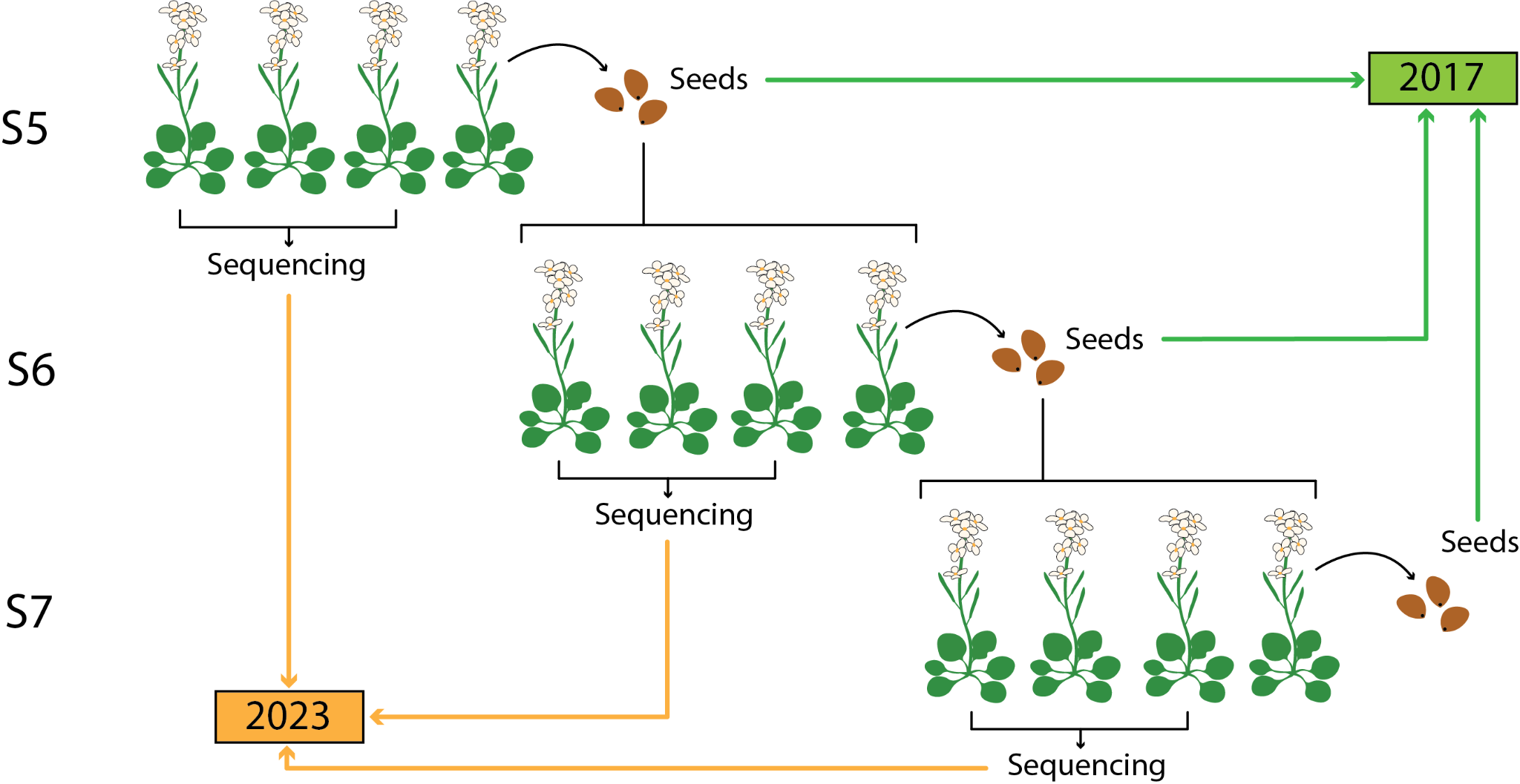


**Figure S1. Illustrative diagram of Arabidopsis sequencing and propagation.** The selfing generations were produced in 2012. Illumina short-read sequencing was performed for every generation in 2023. The plants that produced the seed used for the next generation were no longer available and therefore could not be sequenced. Instead sibling plants were grown and sequenced in 2023.

File S1. Primers for novel junction validation.

File S2. Validated novel DNA junctions.

File S3. Breakpoints origin confirmation through SNPs on sequencing reads.

File S4. Genes directly affected by breakpoints.
